# Supplementary figures and images for: Trends and key disparities of obesity among US adolescents: The NHANES from 2007 to 2020
Source: PLoS One. 2024 Oct 9;19(10):e0290211. doi: 10.1371/journal.pone.0290211 (PMC11463737; doi:10.1371/journal.pone.0290211)

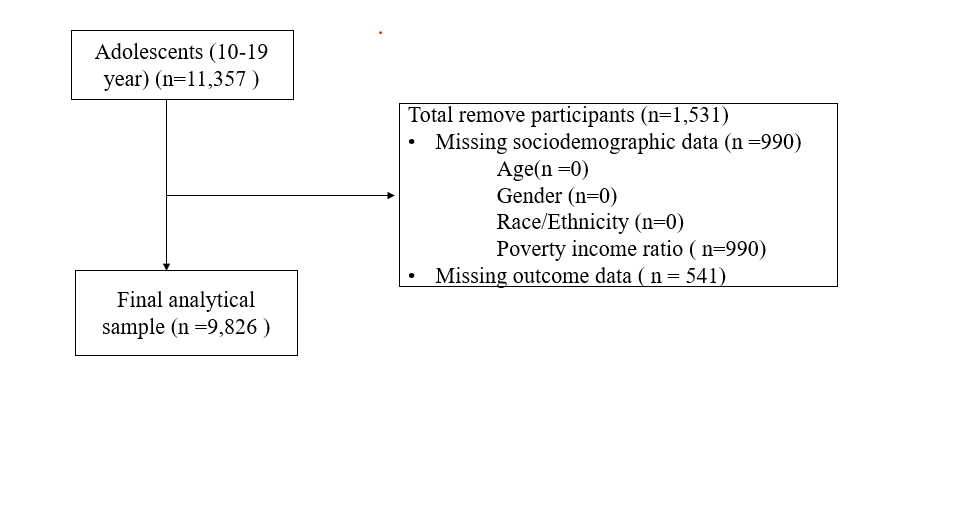


S1 Fig. Sample size flowchart

Supplement: S1 Fig — (DOCX) [file pone.0290211.s001.docx]
