# Supplementary material for: Trends and key disparities of obesity among US adolescents: The NHANES from 2007 to 2020
Source: PLoS One. 2024 Oct 9;19(10):e0290211. doi: 10.1371/journal.pone.0290211 (PMC11463737; doi:10.1371/journal.pone.0290211)
Supplement: S2 Fig — (DOCX) [file pone.0290211.s002.docx]

S2 Fig. Trends in mean BMI among US adults (aged 10-19), NHANES 2007-2020 by (a) Age, (b) Sex, (c) Race/ethnicity, and (d) Poverty income ratio.
