# Supplementary material for: Trends and key disparities of obesity among US adolescents: The NHANES from 2007 to 2020
Source: PLoS One. 2024 Oct 9;19(10):e0290211. doi: 10.1371/journal.pone.0290211 (PMC11463737; doi:10.1371/journal.pone.0290211)
Supplement: S1 Table — (DOCX) [file pone.0290211.s003.docx]

S1 Table. BMI Changes in adolescents aged 10-19 years old, overall analysis and analyses stratified by age, sex, race/ethnicity, and PIR from 2007 to 2020 (n = 9,826)

| **Characteristics** | N (BMI weighted mean, 95% CI)^a^ | | | | | | | |  |
| --- | --- | --- | --- | --- | --- | --- | --- | --- | --- |
|  | All  (n = 9,826) | 2007-2008  (n = 1,455) | 2009-2010  (n = 1,546) | 2011-2012  (n = 1,468) | 2013-2014  (n = 1,671) | 2015-2016  (n = 1,507) | 2017-2020  (n = 2,179) | **P value for**  **trend**^b^ | **Wald test**^c^ |
| **Overall** | 23.23  (23.03, 23.43) | 22.85  (22.40, 23.29) | 23.03  (22.62, 23.45) | 23.15  (22.65, 23.64) | 23.41  (22.87, 23.95) | 23.20  (22.57, 23.83) | 23.59  (23.15, 24.03) | <0.05 |  |
| **Age (%)** |  |  |  |  |  |  |  |  |  |
| 10-14 | 21.96  (21.75, 22.17) | 21.79  (21.31, 22.26) | 21.74  (21.27, 22.21) | 22.03  (21.47, 22.58) | 21.96  (21.45, 22.48) | 21.90  (21.26, 22.53) | 22.35  (21.80, 22.82) | ≥0.05 | ≥0.05 |
| 15-19 | 25.23  (24.92, 25.55) | 24.48  (23.84, 25.13) | 25.13  (24.62, 25.65) | 25.03  (24.79, 26.60) | 25.69  (24.79, 26.60) | 25.56  (24.87, 26.24) | 25.53  (24.94, 26.12) | <0.001 |  |
| **Sex (%)** | | | | | | | | |  |
| Boys | 23.10  (22.85, 23.34) | 22.79  (22.23, 23.34) | 23.11  (22.46, 23.76) | 23.05  (22.62, 23.49) | 23.34  (22.87, 23.82) | 22.92  (22.88, 24.15) | 23.40  (22.88, 23.92) | ≥0.05 | ≥0.05 |
| Girl | 23.30  (23.04, 23.35) | 22.91  (22.33, 23.50) | 22.95  (22.49, 23.41) | 23.23  (22.44, 24.03) | 23.48  (22.67, 24.30) | 23.48  (22.81, 24.15) | 23.78  (23.23, 24.33) | ≥0.05 |  |
| **Race/Ethnicity (%)** | | | | | | | | |  |
| White (%) | 22.77  (22.48, 23.05) | 22.55  (21.97, 23.12) | 22.70  (21.98, 23.41) | 22.72  (22.00, 23.44) | 23.18  (22.34, 24.02) | 22.34  (21.78, 22.90) | 23.22  (22.47, 23.98) | ≥0.05 | <0.001 |
| Black (%) | 24.28  (23.86, 24.69) | 23.90  (23.28, 24.53) | 24.56  (23.79, 25.33) | 24.24  (23.11, 25.38) | 23.72  (22.34, 24.0 2) | 24.47  (22.89, 26.02) | 25.92  (24.28, 25.56) | <0.001 |  |
| Hispanic (%) | 23.94  (23.91, 24.17) | 22.54  (22.89, 24.18) | 23.48  (23.01, 23.96) | 23.42  (22.76,24.08) | 24.01  (23.64, 24.39) | 24.80  (24.16, 25.45) | 24.07  (23.31, 24.83) | <0.001 |  |
| Other race (%)^d^ | 22.57  (22.05, 23.10) | 21.28  (19.84, 22.72) | 21.51  (20.40, 22.62) | 23.39  (21.41, 25.36) | 22.89  (21.68, 23.95) | 22.99  (21.82, 24.16) | 22.72  (22.07, 23.28) | ≥0.05 |  |
| **Poverty Income Ratio** | | | | | | | | |  |
| PIR ≥3.5  (High-income) | 22.16  (21.82, 22.50) | 22.20  (21.59, 22.81) | 22.23  (21.44, 23.03) | 22.07  (21.12, 23.02) | 21.96  (20.73, 23.18) | 21.99  (21.29, 22.70) | 22.47  (21.87, 23.06) | ≥0.05 | <0.001 |
| PIR 1.3 to <3.5  (Middle-income) | 23.39  (23.05, 23.74) | 22.84  (22.10, 23.59) | 22.76  (22.1, 23.37) | 23.80  (22.85, 24.75) | 23.85  (22.74, 24.95) | 23.39  (22.62, 24.16) | 23.67  (22.96, 24.38) | <0.001 |  |
| PIR <1.3  (Low-income) | 24.05  (23.80, 24.30) | 23.57  (22.83, 24.31) | 24.27  (23.81,24.73) | 23.39  (22.90, 23.89) | 24.18  (23.52, 24.84) | 24.28  (23.68, 24.87) | 24.82  (24.22, 25.42) | <0.001 |  |

^a^ Data were weighted to be nationally representative.

^b^A survey-weighted linear regression model was used to evaluate BMI trends, both overall and by the subgroups (age, sex, race/ethnicity, and PIR), 2007 through 2020

^c^A survey-weighted Wald test for an interaction term between survey cycle and sociodemographic factors such as age, sex, race/ethnicity, and PIR

^d^ Other race include individuals self-identifying as non-Hispanic Asian, Other, or being from more than one race or ethnic group.
