# Supplementary material for: Trends and key disparities of obesity among US adolescents: The NHANES from 2007 to 2020
Source: PLoS One. 2024 Oct 9;19(10):e0290211. doi: 10.1371/journal.pone.0290211 (PMC11463737; doi:10.1371/journal.pone.0290211)
Supplement: S2 Table — (DOCX) [file pone.0290211.s004.docx]

S2 Table. Prevalence of obesity in adolescents aged 10-19 years old, overall analysis and analyses stratified by age, sex, race/ethnicity, and PIR from 2007 to 2020 (n = 2,346)

| **Characteristics** | N (obesity prevalence weighted %, 95% CI)^a^ | | | | | | | |  |
| --- | --- | --- | --- | --- | --- | --- | --- | --- | --- |
| Prevalence | All  (n = 2,346) | 2007-2008  (n =341) | 2009-2010  (n = 347) | 2011-2012  (n = 329) | 2013-2014  (n = 385) | 2015-2016  (n = 361) | 2017-2020  (n = 583) | **P value for**  **trend**^b^ | **Wald test**^c^ |
|  | **No. of participants** | **Prevalence**  **(95% CI)** | **Prevalence**  **(95% CI)** | **Prevalence**  **(95% CI)** | **Prevalence**  **(95% CI)** | **Prevalence**  **(95% CI)** | **Prevalence**  **(95% CI)** |  |  |
| **Overall** | 2,346 | 20.71  (17.16, 24.27) | 20.43  (18.21, 22.66) | 21.90  (18.86, 24.94) | 22.94  (19.69, 26.19) | 21.92  (17.70, 26.13) | 23.99  (21.31, 26.67) | <0.05 |  |
| **Age (%)** | | | | | | | | |  |
| 10-14 | 1,572 | 22.02  (18.12, 25.93) | 20.61  (17.85, 22.79) | 23.75  (19.81, 27.68) | 22.23  (18.75, 29.34) | 21.83  (17.04, 27.10) | 25.19  (21.79, 28.59) | ≥0.05 | ≥0.05 |
| 15-19 | 774 | 18.70  (13.25, 24.15) | 20.62  (16.98, 24.25) | 18.77  (11.64, 25.91) | 24.05  (18.69, 25.78) | 22.07  (17.44, 26.22) | 22.11  (18.29, 25.94) | ≥0.05 |  |
| **Sex (%)** |  |  |  |  |  |  |  |  |  |
| Boys | 1,230 | 21.93  (17.47, 26.39) | 22.56  (18.30, 26.81) | 22.56  (18.70, 26.43) | 23.93  (19.87, 27.99) | 23.22  (17.80, 28.66) | 25.67  (22.65, 28.68) | <0.01 | <0.05 |
| Girls | 1,116 | 19.37  (15.46, 23.29) | 18.24  (16.08, 20.41) | 21.20  (17.47, 24.5) | 21.91  (17.40, 26.41) | 20.55  (16.68, 24.41) | 22.35  (18.47, 26.22) | <0.01 |  |
| **Race/Ethnicity (%)** | | | | | | | | |  |
| White (%) | 624 | 18.70  (13.47, 23.93) | 18.19  (14.6, 22.21) | 19.94  (15.34, 24.55) | 21.81  (15.80, 27.82) | 16.38  (12.47, 20.29) | 21.23  (17.05, 25.41) | >0.05 | <0.001 |
| Black (%) | 665 | 24.96  (21.47, 28.45) | 27.76  (22.07, 33.45) | 25.72  (20.23, 31.22) | 22.64  (17.04, 28.23) | 30.47  (21.32, 38.43) | 30.62  (27.18, 34.06) | <0.001 |  |
| Hispanic (%) | 817 | 25.10  (21.51, 28.67) | 24.21  (20.78, 27.62) | 24.34  (19.14, 29.55) | 26.61  (22.71, 30.51) | 28.88  (25.62, 35.31) | 29.05  (22.97, 35.13) | <0.001 |  |
| Other race (%)^d^ | 240 | 18.23  (10.3, 25.6) | 13.88  (7.80, 19.96) | 22.08  (16.45, 27.71) | 21.25  (14.57, 27.94) | 24.31  (12.62, 36.01) | 18.03  (13.28, 22.77) | ≥0.05 |  |
| **Poverty Income Ratio** | | | | | | | | |  |
| PIR ≥3.5  (high-income) | 350 | 17.31  (12.51, 22.21) | 14.84  (10.0, 20.0) | 13.98  (8.28, 19.68) | 15.67  (7.94, 23.38) | 15.48  (11.29, 19.67) | 14.44  (11.50, 17.38) | <0.01 | <0.001 |
| PIR 1.3 to <3.5 (middle-income) | 873 | 19.25  (13.37, 25.12) | 20.52  (16.91, 24.14) | 27.50  (22.54, 32.46) | 25.91  (19.34, 32.48) | 22.95  (18.46, 27.43) | 25.45  (21.32, 29.58) | <0.001 |  |
| PIR <1.3  (low-income) | 1,123 | 26.05  (21.50, 30.61) | 26.67  (24.25, 29.10) | 22.92  (24.25, 29.10) | 25.91  (22.39, 29.43) | 27.60  (22.06, 33.14) | 33.29  (28.47, 38.10) | <0.001 |  |

^a^ Data were weighted to be nationally representative.

^b^A survey-weighted Poisson regression model was used to evaluate the prevalence of obesity trends. both overall and by the subgroups (age, sex, race/ethnicity, and PIR), 2007 through 2020

^c^A survey-weighted Wald test for an interaction term between survey cycle and sociodemographic factors such as age, sex, race/ethnicity, and PIR

^d^ Other race include individuals self-identifying as non-Hispanic Asian, Other, or being from more than one race or ethnic group.
